# Pancreaticopleural fistula: An insidious cause of pleural effusion –case report

**DOI:** 10.34172/jcvtr.2021.37

**Published:** 2021-09-06

**Authors:** Fábio Murteira, Tiago Costa, Sara Barbosa Pinto, Elsa Francisco, Ana Catarina Gomes

**Affiliations:** ^1^Internal Medicine Departement, Centro Hospitalar Vila Nova de Gaia/Espinho, Vila Nova de Gaia, Portugal; ^2^General Surgery Departement, Centro Hospitalar Vila Nova de Gaia/Espinho, Vila Nova de Gaia, Portugal; ^3^Gastroenterology Departement, Centro Hospitalar Vila Nova de Gaia/Espinho, Vila Nova de Gaia, Portugal

**Keywords:** Pancreaticopleural Fistula, Pleural Effusion, Pancreatic Pseudocyst, Pancreatitis, Cholangiopancreatography

## Abstract

Pancreaticopleural fistulas (PPF) are a rare etiology of pleural effusions. We describe a case of a 61-year-old man, with left chest pain with six months of progression who presented with a large volume unilateral pleural effusion. A thoracentesis was performed, which showed a dark reddish fluid(exudate) and high content of pancreatic amylase. After that an abdominal computed tomography (CT)and magnetic resonance cholangiopancreatography (MRCP) was done, revealing fistulous pathways that originated in the pancreas. The patient was admitted for conservative and endoscopic treatment by Endoscopic Retrograde Cholangiopancreatography (ERCP) and a prosthesis was placed on a fistulous path. He was discharged without complications, with the resolution of the pleural effusion and fistula.The interest of this case lies in the rarity of the event and absence of symptoms of the probable primary event (acute pancreatitis). The possible iatrogenic association with several drugs of his usual medication makes it even more complex.

## Introduction

Pancreaticopleural fistulas (PPF) are rare complications of acute or chronic pancreatitis (approximately in 5%)^1^ and are an even less frequent cause of pleural effusion ( < 1%).^2^ They occur more regularly in men in the 5th and 6th decade of life with chronic alcoholism.Despite this, any etiologic mechanism of pancreatitis can be associated with fistulization.The fistula usually occurs due to rupture or malformation of a pancreatic pseudocyst, being the rupture of a pancreatic duct a less common mechanism. Although the primary abdominal repercussion, the most frequent symptoms are respiratory.^3^ Most commonly they cause unilateral pleural effusions ( > ¾ on the left side) with high concentrations of amylase and pancreatic lipase.^4^ The most informative imaging exams are computed tomography (CT), magnetic resonance cholangiopancreatography (MRCP) and endoscopic retrogade cholangiopancreatography (ERCP).^3^ As a rule, treatment initially passes through drainage of the pleural fluid, parenteral feeding and decreasing pancreatic secretion (somatostatin analogs).^4^ It can also be advantageous to use ERCP as a therapeutic weapon (placement of stents in fistulous pathways). Only when this is not effective or the endoscopic route is technically impossible, it is decided to proceed to the surgical intervention.^5^

## Case Presentation

We describe a case of a 61-year-old man, referred to the Emergency Room (ER) with generalized left thoracic pain (six months progression, described as a pressure, without worsening in inspiration, position of relief or worsening). In addition, he reported tiredness in small efforts, anorexia and weight loss (5-10% of total weight). For the mentioned complaints, he had gone to the ER in the first weeks of the condition. At that time, there were no signs of clinical, laboratory or imaging abnormalities and he was discharged without a definite diagnosis; surveillance was indicated.

As pathological history, he had type 2 diabetes mellitus, arterial hypertension and mixed dyslipidemia (13 years of evolution), all of those with a good control in the last two years (usual medication in Table 1). Scarce alcohol use (20-40 g of alcohol / week) was mentioned. His medication history included Fenofibrate (267 mg - 1 dose / day), Chlorthalidone (50 mg - 1 dose / day), Acetylsalicylic acid (150 mg - 1 dose / day), Metformin (1000 mg - 2 doses / day), Atorvastatin (10 mg - 1 dose / day); two months prior to the presentation, he had stopped Empagliflozin (10 mg 1 dose / day) due a medical indication.

**Table 1:** Usual medication on the first visit to the emergency department

**Drugs**	**Dosage**
Fenofibrate	267 mg - 1 dose / day
Chlorthalidone	50 mg - 1 dose / day
Acetylsalicylic acid	150 mg - 1 dose / day
Metformin	1000 mg - 2 doses / day
Atorvastatin	10 mg - 1 dose / day

* Note: He stopped 2 months before Empagliflozin 10 mg 1 dose / day due to medical indication, because his doctor assumed a iatrogenic relationship to the weight loss already presented at that time.

**Figure 1:**
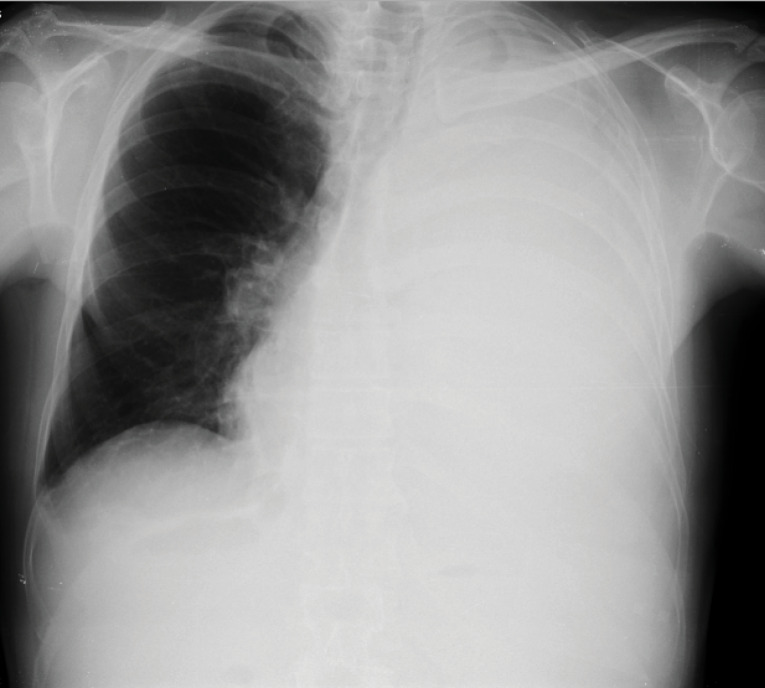
Chest X-ray before the first thoracentesis

In a second visit to the ER the patient had normal vital signs but a marked decrease in breath sounds throughout the left hemithorax. A chest X-ray was performed, which showed homogeneous opacity in the entire left lung and contralateral deviation of the trachea, compatible with large unilateral pleural effusion (Figure 1). Diagnostic and evacuating thoracentesis had been performed (drained a dark reddish liquid). The liquid analysis was compatible with an exudate, with a high number of erythrocytes (130 300/Ul); empyema or parapneumonic effusion was excluded. Bacteriological and mycobacteriological cultural examinations (both negative later) and anatomopathological examination (without malignant cells later) were left ongoing. He was discharged for an early pulmonology follow-up visit.

A week later (before consultation), he returned to the ED due to the reappearance of chest pain. He maintained a large volume pleural effusion, so diagnostic and evacuating thoracentesis was repeated and this time dark brown liquid was drained. On that occasion, pancreatic amylase was added to the analysis, proving to be quite high (2072 U/L). The level of serum of pancreatic amylase (108 U/L) and lipase (116 U/L) were 2 to 3 times above the reference value; transaminase value, liver function parameters and lipid profile (including triglycerides) were all normal.

**Figure 2:**
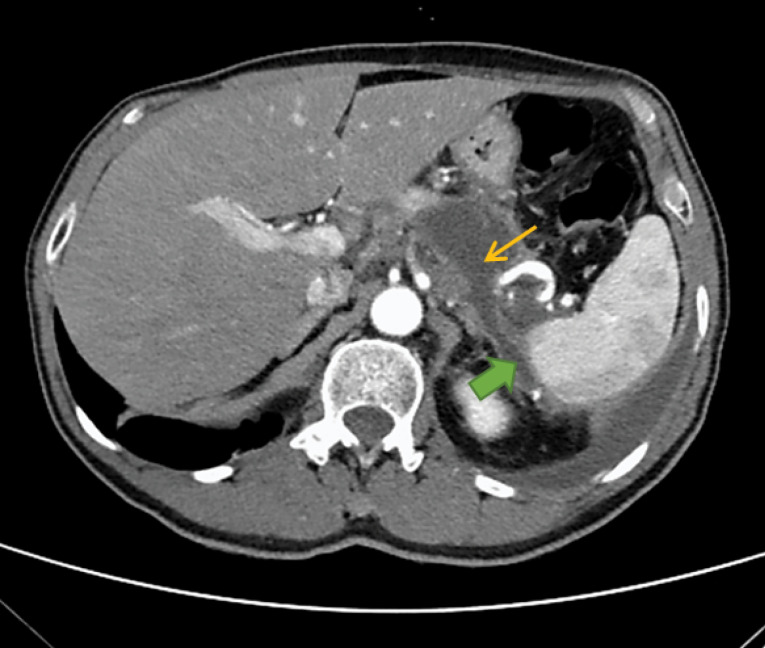
Abdominal Computed Tomography (CT) at the time of diagnosis, with evidence of pancreatic pseudocyst (thin yellow arrow) and one of the fistulous paths (full green arrow)

We decided to perform an Abdominal Computed Tomography (CT): at the pancreatic body / tail transition, a collection with 48 x 36 x 46 mm and two drainage paths were observed (Figure 2). Thus, this findings was suggestive of an acute post-pancreatitis pseudocyst with fistulization to the left pleural cavity and peri-splenic region. He was discharged to General Surgery consultation; however, prior to that MRCP was done, that confirmed the PPF (no suspicium of malignancy).

**Figure 3:**
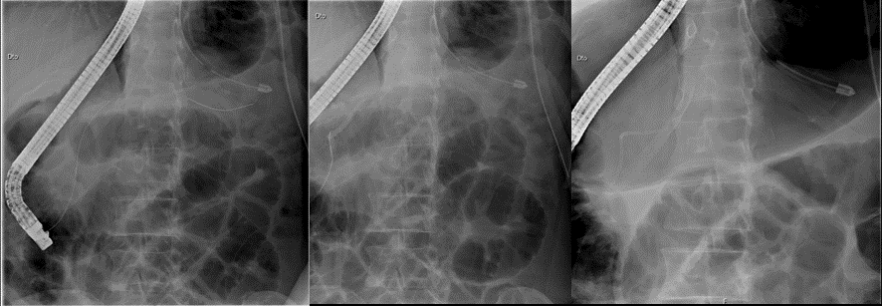
Endoscopic Retrograde Cholangiopancreatography (ERCP) procedure

At that consultation, an elective hospitalization (3 weeks after the first thoracentesis) for pleural drainage, medical care (parenteral feeding and administration of octreotide) and endoscopic (ERCP) treatment was decided. During ERCP, a sphincterotomy was performed and a leak of contrast (high output) was shown in the Wirsung duct; a prosthesis was placed in the proximal region of the evidenced fistulous path (Figure 3). At discharge date (3 days after stent placement), there was no longer significant pleural effusion. The monthly intramuscular octreotide was continued. After three months, the MRCP was repeated, with no sign of pleural effusion and a total resorption of the pseudocyst. With these results a total resolution of the fistulous path was assumed and a new ERCP was scheduled to remove the pancreatic prosthesis.

## Discussion

The importance of this case lies in the rarity of the event and presentation. The absence of symptoms (abdominal pain, nausea, vomiting, steatorrhea) of the probable primary event (acute pancreatitis) preceding chest pain makes it more peculiar. Therefore, it is not possible to specify the beginning of the process or the etiology of the probable acute pancreatitis that caused this complication (PPF). Although the most frequent causes (lithiasis, alcoholism and uncontrolled hypertriglyceridemia) have been excluded, it is difficult to define if pancreatitis was iatrogenic or not. One of the drugs possibly implicated, Empagliflozin (oral antidiabetic drug of the sodium-glucose 2 cotransporter inhibitor - SGLT2i - class), belongs to a class that in recent years has been suspected of having increased risk of pancreatic pathology, with several cases of acute pancreatitis with a possible direct relationship.^6^ Despite this, existing meta-analyses and systematic reviews do not confirm this increased risk.^7,8^ With this patient, a relationship cannot be ruled out (the suspension was already done after the onset of symptoms), but he consumed other drugs occasionally described as a cause of pancreatitis, too.^9^

Although a relevant portion of acute pancreatitis evolves with pleural effusion (up to ¼)^10^ and is an indicator of poor prognosis (20-30% mortality)^11^, in most cases it disappears after the resolution of the acute event.^10^

As in this case, pleural effusion caused by PPFs is more regularly unilateral (mainly left and large volume), with rapid reappearance after evacuation.^3^ As in this case, the levels of amylases of the pleural effusion are frankly elevated (almost always > 1000 U / L).^4^ At the imaging level, it is not always possible to visualize the fistula with CT (MRCP is more sensitive).^3^ In terms of treatment, there is often resolution only with a conservative approach (drainage of the pleural fluid, parenteral feeding and administration of somatostatin analogs, such as Octreotide); these procedures have a success rate of 30-60%.^12^ As with our patient, an endoscopic approach is sometimes decided at the beginning, or it is performed when conservative therapy has not been effective.^4^ ERCP allows a therapeutic intervention through the placement of stents to block the fistulous pathways.^13^ This less invasive method has shown good results (mortality < 5%).^4^ In this patient, there was no need for surgical intervention (more commonly distal pancreatectomy and pancreato-jejunostomy), which is associated with a higher risk of complications.^14^ Until nowadays, there are no clinical, laboratory or imaging parameters that have shown a predictive ability to assess who most benefits from medical, endoscopic or surgical therapy. ^10^ The lack of effective treatment can lead to severe respiratory failure and other complications (such as empyema) with implicated vital risk.^15,16^

## Conclusion

With this case, we intend to draw attention to a complication of abdominal pathology with essentially respiratory manifestations, for which a high level of suspicion is needed, especially in patients with risk factors for acute or chronic pancreatitis.

## Funding

None.

## Ethical approval

The authors have no ethical conflicts to disclose. Informed consent was obtained from the patient.

## Competing interests

None.
